# The association between diet estrogenicity in exotic felids and poor spermatozoa quality in tigers (*Panthera tigris*)^†^

**DOI:** 10.1093/biolre/ioaf161

**Published:** 2025-07-24

**Authors:** Lindsey Fallon, Rachel Felton, Jason Herrick, Taylor VanDeVoorde, Allison Gabel, Christopher W Tubbs

**Affiliations:** Conservation Science Wildlife Health, San Diego Zoo Wildlife Alliance, Escondido, CA, USA; Conservation Science Wildlife Health, San Diego Zoo Wildlife Alliance, Escondido, CA, USA; Department of Reproductive Sciences, Omaha’s Henry Doorly Zoo and Aquarium, Omaha, NE, USA; Conservation Science Wildlife Health, San Diego Zoo Wildlife Alliance, Escondido, CA, USA; Division of Animal Sciences, College of Agriculture, Food, and Natural Resources, University of Missouri, Columbia, MO, USA; Conservation Science Wildlife Health, San Diego Zoo Wildlife Alliance, Escondido, CA, USA; Department of Animal Science, Pennsylvania State University, University Park, PA, USA; Conservation Science Wildlife Health, San Diego Zoo Wildlife Alliance, Escondido, CA, USA

**Keywords:** diet estrogenicity, phytoestrogens, endocrine disruption, spermatozoa morphology, exotic felids

## Abstract

A high incidence of morphologically abnormal spermatozoa has been observed in felid species including snow leopard (SL), Sumatran tiger (ST), and cheetah (CH). In managed settings, these species consume diets different than their wild counterparts, which can contain soy products with detectable levels of phytoestrogens. Previous work has demonstrated that estrogenic diets can interfere with endocrine function and may be associated with reproductive failure. We evaluated the relationship between diet and poor spermatozoa quality in felids by quantifying the estrogenicity of commercial felid diets that were collected from facilities at the time of semen analysis on individual tigers consuming that diet. In vitro estrogen receptor activity was characterized for estrogen receptor alpha (ERα) and estrogen receptor beta (ERβ) of each species by treating with purified estrogens, phytoestrogens, and diet extracts. Phytoestrogens activated all receptors, which were more sensitive to coumestrol and genistein than daidzein or equol at lower concentrations. ST ERβ was the most sensitive to coumestrol and genistein (*P* < 0.05). Of the seven diets tested, three resulted in significant activation of ERβ, two of which contained soy products. The highest levels of activation from each diet occurred on ST ERβ (*P* < 0.05). Significant correlations were found between three tiger spermatozoa defects and diet estrogenicity: bent midpiece (*R*^2^ = 0.2794, *P* = 0.0079), bent midpiece with cytoplasmic droplet (*R*^2^ = 0.1776, *P* = 0.040), and bent tail (*R*^2^ = 0.280, *P* = 0.0078). The potential for dietary estrogenic compounds to contribute to decreased sperm quality may provide novel insight into one reason why morphological abnormalities persist in captive exotic felid spermatozoa.

## Teaser

Estrogenicity of commercial exotic felid diets is associated with decreased spermatozoa quality in tigers under managed care.

## Graphical Abstract

**Graphical Abstract:**
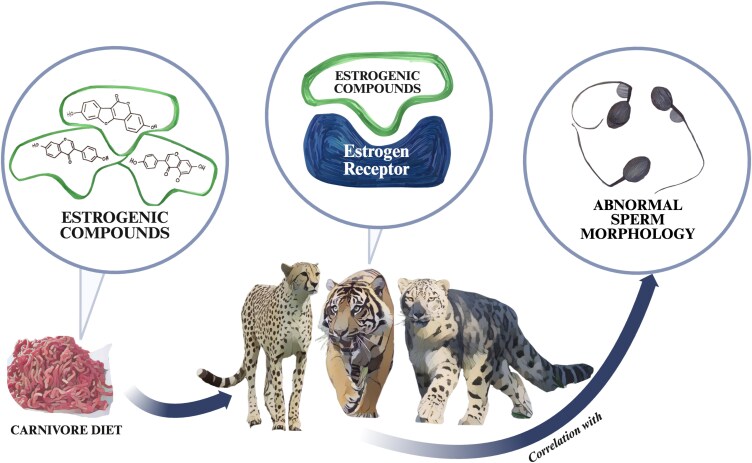

Created in BioRender. Felton, R. (2025). https://BioRender.com/n97s453.

## Introduction

The Felidae family is composed of 38 extant species [1], the majority of which are listed as having decreasing population trends, with 18 currently listed as vulnerable or endangered [2]. The primary reasons for population decline include poaching, illegal wildlife trafficking, habitat loss, and prey depletion [3–6]. These issues continue to impact many wild felid species worldwide, highlighting the need for ex situ populations to serve as genetic reservoirs for diminishing wild populations. Therefore, the importance of ex situ population health and viability is relevant to wildlife conservation in multiple facets.

**Figure 1:**
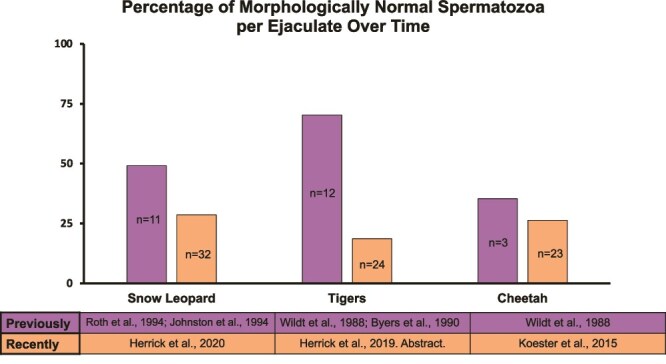
Summary of spermatozoa morphology over time in snow leopards, tigers, and cheetahs.

Some of the earliest studies of reproduction in zoo species involved felids, making them one of the only nondomestic taxa in which 20–30 years of historical data are available on sperm quality [7–9]. Notably, there has been an increase in morphological abnormalities observed per ejaculate in species such as the snow leopard (*Panthera uncia,* SL)— listed as vulnerable, the Sumatran tiger (*Panthera tigris sumatrae*, ST)— listed as endangered, and the cheetah (*Acinonyx jubatus*, CH)— listed as vulnerable (IUCN, 2024), since the 1990s (Figure 1). The morphology of spermatozoa collected from snow leopards in captivity decreased from approximately 49.15% normal [7, 10] to 28.6% normal [11]. In tigers, normal morphology decreased from approximately 70.3% [8, 9] to 18.7% [12]. Lastly, in cheetahs, normal morphology decreased from approximately 35.4% [9] to 26.35% [13]. Across these studies, midpiece defects were most frequently discovered in the spermatozoa, although other prominent defects also included bent or coiled tails and retained cytoplasmic droplets. These defects can have dramatic effects on sperm function in vitro and the ability of the spermatozoa to survive cryopreservation [14]. Therefore, the decline in sperm quality over time could have a pronounced effect on efforts to preserve genetic diversity in genome resource banks [15] and to utilize assisted reproductive technologies for the genetic management of small populations of endangered felids [16].

Individuals of these species in managed settings consume diets that vary from those of their wild counterparts, including meat from livestock species and alternative sources of protein [17]. Commercial exotic feline diets are often fed as the majority of the diet in zoos due to their comprehensive nutritional make-up. Previous literature has demonstrated that many commercial feline diets contain soybean additives [18, 19], as well as detectable levels of phytoestrogens such as genistein and daidzein, which are commonly found in soy and soy-based products [17, 19]. Studies have shown that estrogenic diets potentially interfere with normal endocrine function in other captive exotic species such as the southern white rhinoceros (*Ceratotherium simum simum*) and are associated with sub-fertility and reproductive failure [20, 21]. Additionally, it has been documented that adult male rats being fed a high-phytoestrogen diet (465 mg/g total phytoestrogens, made up of 225 mg/g genistein, 180 mg/g daidzein, and 60 mg/g glycitein) had significantly decreased sperm counts, increased incidence of apoptotic germ cells, and increased incidence of lipid peroxidation of epididymal sperm [22, 23].

One way in which estrogenic compounds can exhibit negative physiological impacts is by interacting directly with estrogen receptors (ERs), leading to endocrine disruption. It is known that both estrogen receptor alpha (ERα) and estrogen receptor beta (ERβ) are expressed in the testes of several mammalian species [24–26]. Additionally, endogenous estrogens are produced at the site of sperm production in mammals and male mice that lack functional ERs are infertile. Adult male estrogen receptor knock-out (ERKO) mice develop severe abnormalities in seminiferous tubule morphology, produce significantly less viable spermatozoa, and fail to achieve fertilization in vitro, suggesting that proper ER signaling is necessary for successful production of spermatozoa [27]. Therefore, investigating the estrogenic activity of exotic felid diets may provide insight into a potential correlation with poor sperm morphology in novel species.

The hypothesis of the current study was that estrogenic compounds present in the diets of captive exotic felids have the ability to activate ERs and act as endocrine disrupters, leading to the production of morphologically abnormal spermatozoa. Here, we aimed to evaluate this relationship by quantifying the estrogenicity of commercial felid diets that were collected from various facilities at the time that semen analysis was performed on individuals consuming that diet. We first characterized the estrogen receptor activity of ERα and ERβ for all three species in vitro*,* when treated with purified endogenous estrogens and phytoestrogens. Next, following the collection of the diets and semen analyses, we developed and validated an effective method to extract estrogenic compounds from meat diets, allowing us to treat all receptors with diet sample extracts and assess their ability to achieve ER activation. Finally, we evaluated whether there was a discernible relationship between spermatozoa morphology and the estrogenicity of commercial diets fed to tigers during the time of semen collection and evaluation.

## Materials and methods

### Animals

Male tigers ranging from 2.7 to 17.2 years old were maintained at zoos accredited by the Association of Zoos and Aquariums (AZA). Procedures were approved by each institution’s Research Review Committee and/or Animal Care and Use Committee. Although diets were often supplemented with whole prey items (e.g., rabbit carcasses) and/or enrichment items (e.g., oxtails), the majority of all diets were composed of the commercial carnivore diets collected and subjected to analysis. All individuals were consuming sampled diets for a minimum of 1 month prior to collection. Collections were opportunistic and were not assigned to control or treatment groups.

Anesthesia was administered and monitored throughout the procedures by the attending veterinarians at each institution. All male tigers were immobilized with ketamine (3.3 ± 0.2 mg/kg) and medetomidine (0.032 ± 0.002 mg/kg), with some also receiving midazolam (0.10 ± 0.01 mg/kg, *n* = 23 procedures) and/or butorphanol (0.11 ± 0.1 mg/kg, *n* = 9 procedures). In some cases (*n* = 16 procedures), isoflurane was administered to maintain anesthesia throughout the duration of the procedure.

### Semen collection and analysis

Testes were measured with calipers to determine testicular volume (cm^3^, length * width^2^ * 0.524) [28]. The penis was manually extruded from the prepuce and examined for normal morphology and gently cleaned of any debris using moist gauze. Semen collection was performed using a standardized electroejaculation procedure that has been validated for safety and efficacy in numerous felid species, including tigers [11]. Briefly, a lubricated probe containing three ventral electrodes was inserted into the rectum to align with the pelvic rim and accessory glands. A controlled series of stimuli was applied in three sets of 10 stimuli with increasing voltage for each set. The male was allowed to rest 5–10 min after each series of 30 stimuli, and the procedure was repeated twice for a total of 90 stimuli (3–6 V per stimuli). Semen was collected into warmed, sterile 50 mL conical tubes (BD Falcon) that were held in place over the extruded penis. At the conclusion of each series, the pH of the fluid was determined with indicator strips (pH Indicator Strip; Millipore Sigma, St. Louis, MO) and the volume of ejaculate was measured to the nearest μl with a pipettor as it was transferred to a fresh polystyrene tube. Motility was evaluated by placing a small aliquot (5 μl) on a warmed glass slide and visually assessing at 100×. Remaining samples were diluted 1:1 with HEPES-buffered (2-[4-(2-hydroxyethyl)piperazin-1-yl]ethanesulfonic acid) Feline Optimized Culture Medium [29] and maintained at room temperature until the procedure was completed. At the conclusion of the procedure, the samples from each series were combined, the sperm concentration was determined with a hemocytometer, and the sample was processed for cryopreservation. If urination occurred during the procedure, the concentration of spermatozoa present was determined and used in calculations of sperm recovery, but these samples were discarded. A small aliquot (5–10 μL) of fresh, undiluted seminal fluid was fixed with 50 μL of 0.4% glutaraldehyde for later assessment of sperm morphology. Individual spermatozoa (*n* = 200) were evaluated at 1000× and categorized as normal or exhibiting head, midpiece, or tail abnormalities previously described for felids [28].

### Tissue collection for estrogen receptor cloning

All procedures in this study were approved by San Diego Zoo Wildlife Alliance’s (SDZWA’s) Institutional Animal Care and Use Committee. Felid ovaries were collected at the time of necropsy by SDZWA pathologists, to be used for cloning and identification of species-specific estrogen receptor (ESR) sequences and underwent a slow freeze protocol of cooling 0.3 °C per minute until reaching −40 °C in PBS supplemented with 1.5 M dimethyl sulfoxide (DMSO) prior to being stored in LN2. Storage media was removed by centrifugation and resuspended in 1 mL Trizol (Life Technologies, Carlsbad, CA) per approximately 100 mg tissue. For total RNA extraction, cheetah (SB#7005), snow leopard (SB#2418), and Sumatran tiger (SB#542) ovaries were homogenized (PowerGen125, Fisher Scientific, Pittsburgh, PA) in Trizol prior to extraction. RNA quality was assessed using an RNA 6000 Nano Kit (Agilent Technologies, Santa Clara, CA) to determine the RNA integrity number for each sample (RIN; cheetah 6.8, snow leopard 7.8, Sumatran tiger 7.7) before performing receptor cloning described below.

### Estrogen receptor cloning

Approximately 1 μg of RNA was reverse-transcribed using SuperScript IV Reverse Transcriptase (Invitrogen, Carlsbad, CA). To capture open reading frames, primers (Supplemental Table 1) were designed for start and stop sites of Cat ESRs based on an alignment (Clustal Omega, EMBL-EBI) of predicted felid ESR1 or ESR2 sequences (GenBank). Full length PCR using Platinum PCR Supermix HiFidelity (Life Technologies) was performed and bands 1788 base pairs in length (ESR1) and 1653 base pairs (ESR2) were excised from the gel and cleaned using a gel DNA recover kit (Zymo Research, Irvine, CA). Excised bands were next subcloned into pCR2.1 TA cloning vectors (Life Technologies), transformed into TOP10 competent *Escherichia coli* (Life Technologies) and positive colonies were identified and finally purified with a ZR plasmid miniprep classic kit (Zymo Research). Consensus sequences were confirmed for the three felid species, two receptors each (ESR1 and ESR2; Supplemental Table 1). Prior to synthesis, the receptors’ complexity and secondary structure were reduced using Integrated DNA Technologies (IDT, Coralville, IA) codon optimization tool, resulting in codon-optimized (CO) sequences for all receptors except those with low complexity scores (SLESR2 and CHESR2). Full-length felid ESRs were synthesized and cloned into pcDNA3.1+ expression vectors using IDT’s custom gene synthesis. Plasmids were reconstituted and transformed into TOP10 competent *E. coli*, cultured, and finally purified using a ZymoPureII Maxi Prep Kit (Zymo Research). Receptor sequences were verified by Sanger sequencing by a commercial vendor (Eton Bioscience, San Diego, CA; Supplemental Table 1). Protein sequences were aligned using MuscleWS via Jalview (Jalview, University of Dundee, Dundee, Scotland) and were then run through Protein BLAST (NCBI, Bethesda, MD) to identify domains where differences in amino acids occurred.

### Steroids and phytoestrogens

17β-estradiol (E_2_), estrone (E_1_), and estriol (E_3_) were purchased from Steraloids (Newport, RI) and dissolved in DMSO (Millipore Sigma) to a concentration of 10 mM. Coumestrol, equol, daidzein, and genistein were purchased from Indofine Chemical Company (Hillsborough, NJ) and dissolved in DMSO at a concentration of 100 mM prior to use.

### Diet extractions

**Figure 2:**
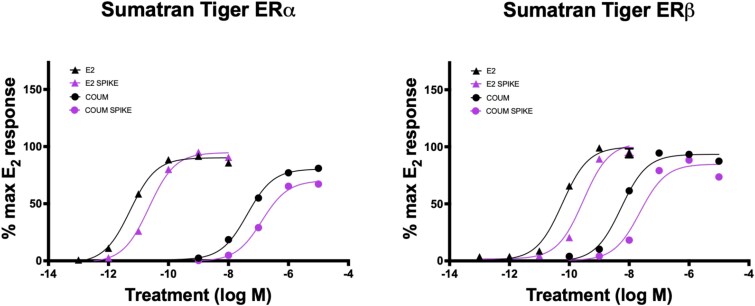
Validation of diet extraction methods. Grassfed beef samples were spiked with either purified estradiol (E_2_) or coumestrol (COUM) prior to extraction and were tested on ERα and ERβ of ST at concentrations of 10^−13^ M – 10^−8^ M of E_2_ or 10^−10^ M – 10^−5^ M of coumestrol. Activation achieved was compared to that of purified E_2_ and coumestrol treatments of respective concentrations, run on the same assay (*n* = 3). Extraction methods were confirmed to effectively recover E_2_ at 104 ± 4.05% and coumestrol at 80.82 ± 2.57% when calculated using ERα activity, and at 96.48 ± 4.41% and 84.73 ± 5.49% when calculated using ERβ activity, respectively.

A total of 24 commercial diet samples were collected from facilities at the time of semen analyses and processed for the extraction of estrogenic compounds. Each diet was aliquoted into 1 g portions (wet weight) in 15 mL conical tubes (Thermo-Fisher Scientific, Carlsbad, CA) prior to beginning the extraction. Laboratory grade methanol (MeOH, Thermo-Fisher Scientific) and sterile water (H_2_O) were added at an 80:20 ratio to each conical tube (3.6 mL MeOH + 0.9 mL H_2_O) along with five sterile, stainless-steel homogenization beads. Tubes were evenly placed into a SPEX SamplePrep 1600 MiniG tissue homogenizer (Cole-Parmer SamplePrep, Metuchen, NJ) to shake at 1500 rpm for 10 min, removed and incubated at −20 ° C for 5 min, and homogenized for an additional 10 min at 1500 rpm to ensure thorough homogenization. Tubes were then centrifuged at 2000× g for 15 min at room temperature. Glass Pasteur pipettes were used to remove the supernatant and transfer to clean 16 × 100 mm glass culture tubes (Thermo-Fisher Scientific). Hypersep C18 500 mg columns (Thermo-Fisher Scientific) were first conditioned with 5 mL pure MeOH, using a HyperSep glass block vacuum manifold (Thermo-Fisher Scientific), followed by conditioning twice with 5 mL sterile H_2_O. Drip rates were maintained at two to four drops per second, and columns were monitored closely to ensure that they did not completely dry. Sterile serological pipette tips were used to transfer the extract supernatant onto the conditioned columns, and a drip rate of two drops per second was maintained using the vacuum manifold. The columns were then washed with 5 mL sterile H_2_O before eluting the hydrophobic compounds with 4.5 mL pure MeOH. For the final elution, a drip rate of two drops per second was maintained until all MeOH was pulled out of the column reservoir; then, vacuum pressure was increased to ensure all MeOH remaining inside the column filter was collected into fresh 16 × 100 mm glass culture tubes. Tubes were placed under a forced air dryer in a fume hood, and the sides of the tubes were rinsed twice with 1 mL pure MeOH during the drying process to ensure the concentration of estrogenic compounds at the bottom of the tubes. Once dry, tubes were capped, sealed with parafilm, and stored at −20 °C until being resuspended with 250 μL DMSO for use in the activation assays (Supplemental Figure 1). The final resuspended concentration of each diet sample was 4 g/mL. Each extraction included a ground grassfed beef sample, a ground grassfed beef sample spiked with a final concentration of 10 mM purified coumestrol, and an empty vehicle tube for controls to ensure efficacy of the extraction. The repeatability of extraction methods was validated by extracting grassfed beef samples spiked with coumestrol or E_2_ in three independent replicates, then treating cells transfected with ST ESR1 and ESR2 in triplicate with dilutions calculated to range from 10^−13^ M – 10^−8^ M of E_2_ or 10^−10^ M – 10^−5^ M of coumestrol, and comparing to the activation achieved when treated with purified E_2_ and coumestrol of respective concentrations, run on the same assay (Figure 2).

### Estrogen receptor activation assays

Receptor activation assays were conducted with SL, ST, and CH ESRs 1 and 2 as described previously [20, 30]. Human embryonic kidney cells (HEK293) were added to 96-well plates in minimum essential medium (MEM) (Corning, Manasses, VA) with 10% fetal bovine serum (FBS) and 1% penicillin–streptomycin (Cytiva Life Sciences, UT). After 24-h incubation at 37 ° C with air containing 5% CO_2_, cells were transfected in MEM with 5 μg pCMX*-*β-galactosidase (β-gal), 5 μg pGL2-3xERE luciferase reporter plasmid (Addgene plasmid 11,354; [31], and 500 ng of the respective ESR-pcDNA3.1(+) expression plasmid per plate using TransIT 2020 transfection reagent (Mirus Bio LLC, Madison, WI). After 24 h, transfected cells were then treated in triplicate with 10^−13^ M – 10^−8^ M of endogenous estrogen, 10^−10^ M – 10^−5^ M phytoestrogen, 0.4–4 mg/mL diet sample or grassfed beef extracts, or a vehicle control treatment of 0.1% DMSO. After 24-h incubation, cells were lysed and assayed to measure luciferase and β-gal activity (Supplemental Figure 1). Luciferase values were normalized to β-gal values, and the fold activation response of each ligand concentration was calculated relative to the vehicle control. The data were then normalized to the maximal activation achieved by each receptor when treated with E_2_, which was run on each assay and calculated by GraphPad Prism (Version 10). The degree of activation by each test compound or diet extract was expressed as a percentage of the sigmoidal dose–response of E_2_ as previously described [30]. All results of ERα were additionally corrected for constitutive receptor activity by subtracting the fold activation of the vehicle from each assay.

### Statistical analyses

All curve fitting and statistical analyses were performed in GraphPad Prism (version 10.4.1; San Diego, CA). Receptor activation data are presented as means ± SEM and were analyzed using a two-way ANOVA and Tukey’s multiple comparisons, with significance set at *P* < 0.05. To ensure repeatability of the assay, all test compounds were run in three independent replicates. Each diet extract was initially tested once, with a subset of diets tested in three independent replicates to further validate the repeatability of the observed results. Comparisons of spermatozoa characteristics and diet estrogenicity were evaluated with the receptor activity of ST ERβ at the highest concentration point of 4 mg/mL and were analyzed using a simple linear regression with significance set at *P* < 0.05.

## Results

### Semen collection and analysis

**Figure 3:**
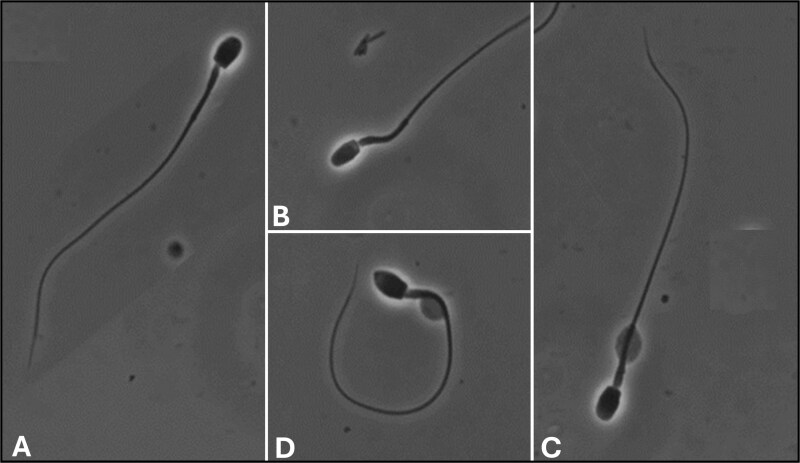
Tiger spermatozoa (phase contrast, 1000×) exhibiting (A) normal morphology, (B) a bent midpiece, (C) a retained cytoplasmic droplet, and (D) a bent midpiece with a retained cytoplasmic droplet and a bent tail.

Semen collections (n = 27) were performed on 24 captive-born male tigers (9 Amur, 5 Malayan, and 10 Sumatran) maintained at 17 zoos (Table 1) from February 2017 to March 2019. The age of individuals ranged from 2.7 to 17.2 years old. Ejaculate volume averaged 6.6 ± 0.7 mL and a pH of 8.4 ± 0.1. Spermic ejaculates were recovered in 26 of 27 collections (214.6 ± 40.6 × 10^6^ spermatozoa per ejaculate), with wide variation between males (0–745.3 million spermatozoa per ejaculate). The majority of spermatozoa were motile (75.2 ± 3.4%), but only 14.9 ± 2.2% exhibited normal morphology (Figure 3). The most commonly observed abnormalities were a proximal cytoplasmic droplet (38.5 ± 4.6%, Figure 3) and a bent midpiece with a cytoplasmic droplet (14.5 ± 1.9%, Figure 3), followed by a bent tail (11.7 ± 1.8%, Figure 3).

**Table 1:** Summary of semen collection procedures performed on male tigers

	**Mean ± SEM** ^**a**^	**Range**
Age (y)	8.8 ± 0.7	2.7–17.2
Body weight (kg)	142.3 ± 5.3	109.3–203.5
Total testicular volume (cm^3^)	62.0 ± 3.4	34.1–106.6
Ejaculate volume (mL)	6.6 ± 0.7	2.0–13.9
Ejaculate pH	8.4 ± 0.1	7.1–8.9
Total spermatozoa recovered (×10^6^)	214.6 ± 40.6	0.0–745.3
Motility (%)	75.2 ± 3.4	30–90
Morphological category (%)		
Normal	14.9 ± 2.2	2.5–44.4
Proximal cytoplasmic droplet	38.5 ± 4.6	6.5–76.5
Bent midpiece w/ cytoplasmic droplet	14.5 ± 1.9	5.6–46.5
Bent tail	11.5 ± 2.1	1–40.5
Tightly coiled tail	6.6 ± 1.8	0–37.5
Bent midpiece	5.4 ± 1.0	0.5–19
Distal cytoplasmic droplet	1.6 ± 0.4	0–6.5

^a^Means represent data from 27 procedures performed on 24 males (*n* = 23 to 27 per endpoint).

### Estrogen receptor cloning

Receptor protein sequences were aligned with one another to identify differences in amino acids for ERα and ERβ of SL, ST, and CH. Overall, ERα was 99.33% similar across all three species and ERβ was 98.73% similar across all three species. In ERα, four differences were identified (Supplemental Figure 2). Amino acid 53, within the A/B domain (activation function region), is a serine (S) in both SL and ST but is an asparagine (N) in CH. Amino acid 76, within the A/B domain, is a serine (S) in both SL and CH but is a proline (P) in ST. Amino acid 496, within the E domain (ligand binding region), is a threonine (T) in SL and ST but is a serine (S) in CH. Amino acid 585, within the F domain, is a threonine (T) in SL and CH but is a serine (S) in ST. In ERβ, seven differences were identified (Supplemental Figure 2). Amino acid 31, within the A/B domain, is a serine (S) in both SL and ST but is a cysteine (C) in CH. Amino acid 40, within the A/B domain, is a serine (S) in SL and ST but is a proline (P) in CH. Amino acid 138, within the A/B domain, is a valine (V) in ST and CH but is an isoleucine (I) in SL. Amino acid 147, within the A/B domain, is an arginine (A) in SL and ST but is a valine (V) in CH. Amino acid 361, within the E domain, is a glycine (G) in SL and CH but is a glutamic acid (E) in ST. Amino acid 433, within the E domain, is an arginine (R) in SL and ST but is a glutamine (Q) in CH. Amino acid 550, within the F domain, is a glutamine (Q) in SL and CH but is a glutamic acid (E) in ST.

### Estrogen receptor characterization with purified compounds

**Figure 4:**
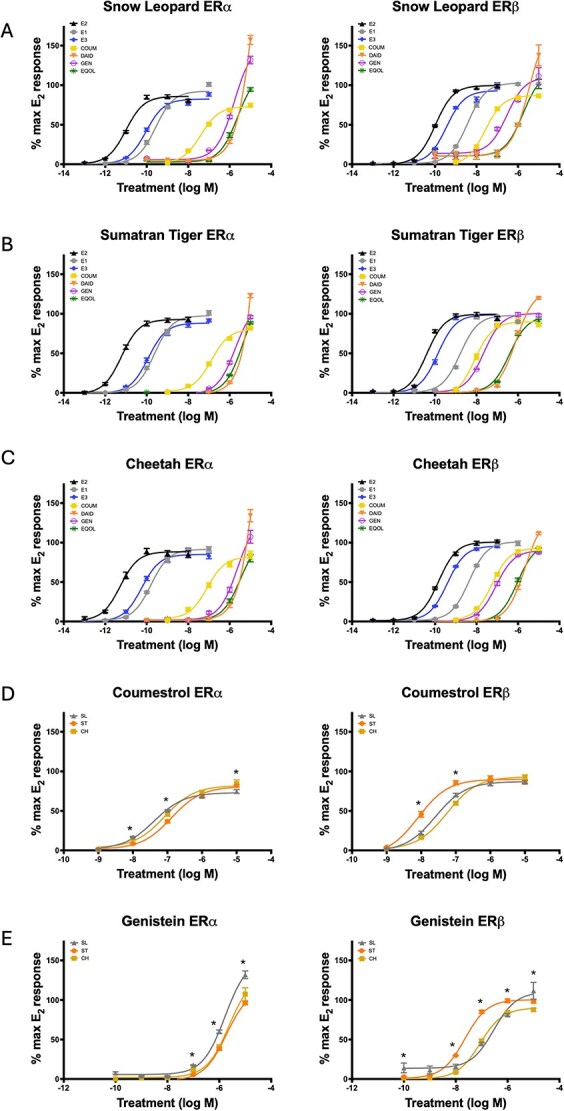
Characterization of SL, ST, CH ERα and ERβ when treated with purified endogenous estrogens (E2 = estradiol, E1 = estrone, E3 = estriol) and phytoestrogens (COUM = coumestrol, DAID = daidzein, GEN = genistein, EQOL = equol) (A–C, *n* = 3). Species differences when treated with coumestrol and genistein (D, E, *P* < 0.05, *n* = 3).

When treated with purified endogenous estrogens and phytoestrogens, both ERα and ERβ of each species (SL, ST, CH) achieved activation (Figure 4), but there were differences in the sensitivities between the receptor type and species for various compounds. For all three species, E_2_ was the most potent agonist of both ERα and ERβ. The concentrations required to reach the half maximal effective concentration (EC_50_) for ERα were 9.693 × 10^−12^ for SL, 6.106 × 10^−12^ for ST, and 5.385 × 10^−12^ for CH. For ERβ, EC_50_ values were 9.808 × 10^−11^ on SL, 4.104 × 10^−11^ on ST, and 1.349 × 10^−10^ for CH. When treated with purified phytoestrogens, all felid receptors were more sensitive to coumestrol and genistein than either daidzein or equol at lower concentrations (Table 2). ST ERα was less sensitive to coumestrol than SL or CH ERα (EC_50_ = 1.305 × 10^−7^ for ST, 4.13E × 10^−8^ for SL, and 8.224 × 10^−8^ for CH). Conversely, ST ERβ was the most sensitive of the three species when treated with coumestrol and genistein (EC_50_ of coumestrol = 8.169 × 10^−9^ for ST, 2.601 × 10^−8^ for SL, and 5.58 × 10^−8^ for CH; EC_50_ of genistein = 2.23 × 10^−8^ for ST, 2.91 × 10^−7^ for SL, and 8.73 × 10^−8^ for CH). Furthermore, ST ERβ achieved significantly higher activation at coumestrol concentrations of 10^−8^ M to 10^−7^ M than either other SL or CH (*P* < 0.05, Figure 4) and at genistein concentrations of 10^−8^ M to 10^−6^ M (*P* < 0.05, Figure 4).

**Table 2:** Summary of the activation of SL, ST, and CH ERα and ERβ when treated with purified phytoestrogens. Results show the half maximal effective concentration (EC_50_) and the mean ± SEM of the maximum activation achieved by each receptor (*n* = 3)

	**SL ER⍺**			**ST ER⍺**			**CH ER⍺**	
	EC50 (M)	Maximum (% max E2)		EC50 (M)	Maximum (% max E2)		EC50 (M)	Maximum (% max E2)
**Phytoestrogen**								
Coumestrol	4.13 × 10^−8^	74.56 ± 2.44		1.31 × 10^−7^	81.76 ± 1.75		8.22 × 10^−8^	84.57 ± 4.71
Genistein	1.67 × 10^−6^	131.82 ± 4.80		1.89 × 10^−6^	95.94 ± 1.89		2.34 × 10^−6^	107.39 ± 8.15
Daidzein	1.51 × 10^−5^	157.25 ± 5.67		7.32 × 10^−5^	122.97 ± 2.52		1.51 × 10^−5^	118.66 ± 1.33
Equol	2.42 × 10^−6^	94.61 ± 2.27		4.70 × 10^−6^	88.51 ± 1.50		3.27 × 10^−6^	80.00 ± 4.46
	**SL ERβ**			**ST ERβ**			**CH ERβ**	
	EC50 (M)	Maximum (% max E2)		EC50 (M)	Maximum (% max E2)		EC50 (M)	Maximum (% max E2)
**Phytoestrogen**								
Coumestrol	2.60 × 10^−8^	86.56 ± 1.70		8.17 × 10^−9^	86.18 ± 2.23		5.58 × 10^−8^	92.69 ± 2.90
Genistein	2.91 × 10^−7^	111.67 ± 10.57		2.23 × 10^−8^	97.94 ± 1.93		8.73 × 10^−8^	87.75 ± 1.83
Daidzein	3.40 × 10^−6^	137.66 ± 13.26		7.93 × 10^−7^	119.58 ± 1.39		2.21 × 10^−6^	111.55 ± 1.61
Equol	1.76 × 10^−6^	101.59 ± 5.89		3.87 × 10^−7^	90.61 ± 2.60		9.40 × 10^−7^	90.53 ± 2.53

### Estrogenicity of diets

**Figure 5:**
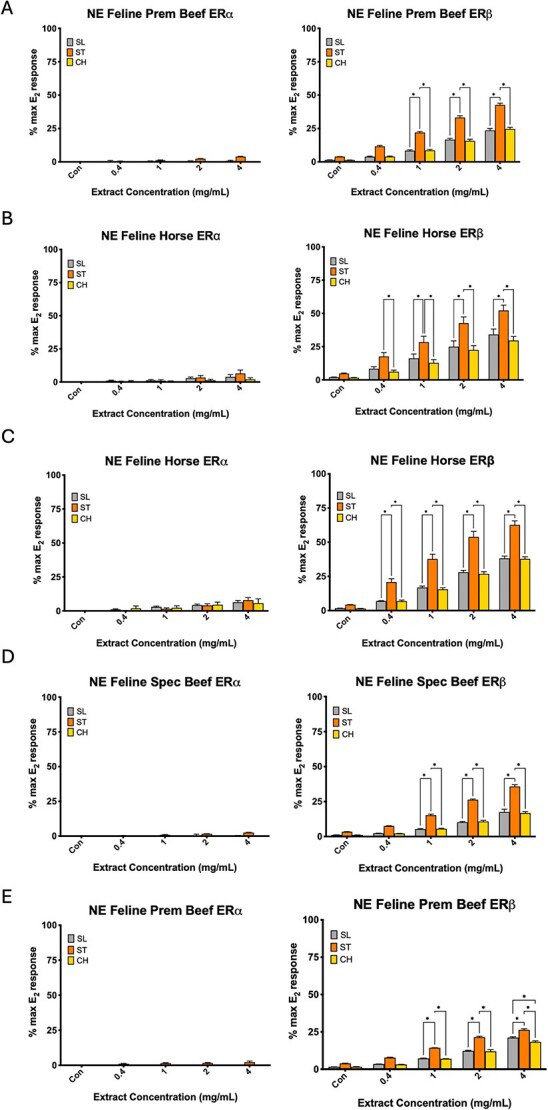
Examples of ER activation by diet samples collected from various institutions (A–E). Results are represented as mean ± SEM of the fold activation of each diet (*P* < 0.05). (A–C) Three independent replicates were run. (D, E) One replicate was run. All significant comparisons shown are also significantly higher than control activation (*P* < 0.05). ST was more sensitive than SL or CH to each of the four diets. Additionally, each diet achieved higher activation on ERβ than ERα.

**Figure 6:**
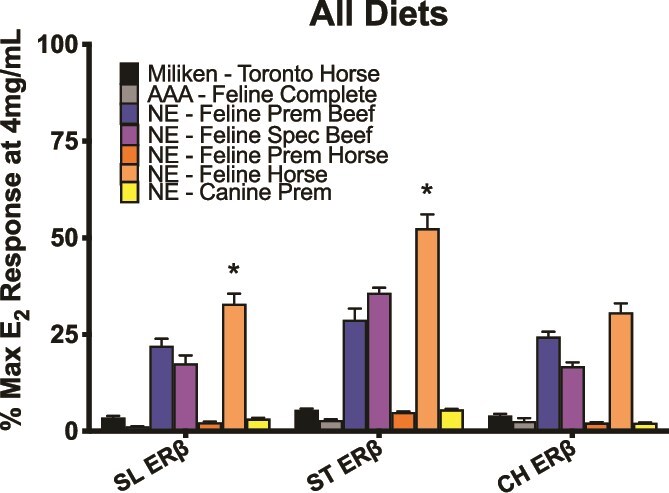
Activation achieved by each diet type on ERβ of SL, ST, and CH across all diet samples of the study. NE—Feline Horse achieved the highest level of activation *(P* < 0.05)*,* followed by NE—Feline Prem. Beef and NE—Feline Spec. Beef.

Diet extraction methods were confirmed to effectively recover E_2_ at 104 ± 4.05% and coumestrol at 80.82 ± 2.57% (Figure 2). Seven commercial diet formulations were collected from 17 AZA-accredited zoological institutions at the time of semen collections. Two facilities were feeding different diets to different males that were collected, resulting in a total of 24 diet samples to be tested for estrogenic activity (Supplemental Table 2). The following diet formulations were tested: Milliken—Toronto Horse, AAA—Feline Complete, NE—Feline Prem Beef, NE—Feline Spec Beef, NE—Feline Prem Horse, NE—Feline Horse, and NE—Canine Prem. Across all diets tested, estrogenic activity was more frequently measured on ERβ than ERα for each of the three species (Figures 5 and 6). Although one sample of NE—Feline Prem Beef achieved significant levels of activation on ERα of each of the three species, with ST ERα reaching significantly higher levels of activation than SL or CH (*P* < 0.05, not shown). Additionally, for all diet samples that achieved activation on ERβ, ST was consistently more sensitive and reached significantly higher levels of activation than SL or CH (*P* < 0.05, Figures 5 and 6). When grouped by diet type across all collections, NE—Feline Horse had the highest estrogenic activity compared to all other diets, which was significant on ST and SL ERβ (*P* < 0.05, Figure 6). Following NE—Feline Horse, both NE—Feline Prem Beef and NE—Feline Spec Beef achieved high levels of activation on ERβ of each of the three species, although which of these two diets achieved higher activation varied by species (Figure 6).

### Spermatozoa morphology and diet estrogenicity

**Figure 7:**
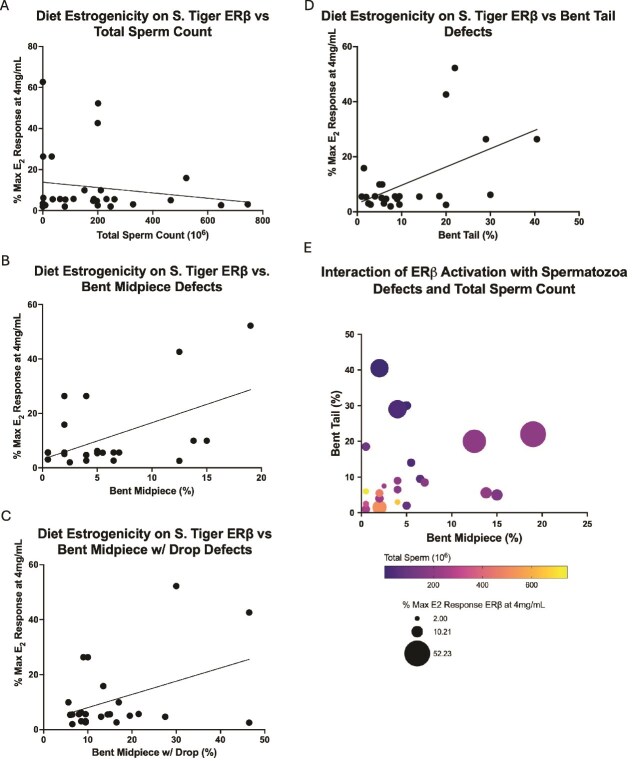
Spermatozoa quality and diet estrogenicity when evaluated with the receptor activity of ST ERβ at 4 mg/mL. (A) No correlation was observed between diet estrogenicity and total sperm count (*R*^2^ = 0.0264, *P* = 0.3997). (B–D) Significant correlations were observed between diet estrogenicity and bent midpiece (*R*^2^ = 0.2794, *P* = 0.0079), bent midpiece with cytoplasmic droplet (*R*^2^ = 0.1776, *P* = 0.040), and bent tail (*R*^2^ = 0.280, *P* = 0.0078). (E) Multiple variable plot illustrating the interaction of ERβ activation and spermatozoa characteristics.

No significant correlation was observed between diet estrogenicity and the number of spermatozoa recovered per ejaculate (*R*^2^ = 0.0264, *P* = 0.3997, Figure 7) or the body weight of individuals and the number of spermatozoa (*R*^2^ = 0.0544, *P* = 0.2234, not shown). Significant correlations were found between the prevalence of three morphological defects and diet estrogenicity: bent midpiece (*R*^2^ = 0.2794, *P* = 0.0079, Figure 7), bent midpiece with a cytoplasmic droplet (*R*^2^ = 0.1776, *P* = 0.040, Figure 7), and bent tail (*R*^2^ = 0.280, *P* = 0.0078, Figure 7). Age at the time of collection did not have a significant effect on bent midpiece (*R*^2^ = 0.0086, *P* = 0.6658 Supplemental Figure 3), bent midpiece with a cytoplasmic droplet (*R*^2^ = 0.0430, *P* = 0.3312, Supplemental Figure 3), or bent tail (*R*^2^ = 0.0954, *P* = 0.1421, Supplemental Figure 3).

## Discussion

This study evaluated the estrogenic activity of commercial felid diets when tested on ERα and ERβ of SL, ST, and CH. Each of these species has experienced a decline in morphologically normal spermatozoa over the last 30 years (Figure 1), complicating their ability to reproduce in managed settings and maintain sustainable populations. Therefore, it is imperative that research continues to investigate potential contributions to abnormal sperm production. Tigers had the most notable decline in spermatozoa quality during this timeframe [8, 9, 29] and were the targeted species for this study’s paired diet and semen collections. One limitation of this study design was the inability to achieve serial collections of diet and semen samples, preventing a longitudinal analysis from being performed. Although a repeated semen collection was only possible for three individuals (two animals at one institution and one animal at another institution; Supplemental Table 2), this data set includes 21.43% of male tigers (24/112 individuals) within the AZA regional studbook for North America and remains valuable.

The present data determined that SL, ST, and CH ERs achieve significant levels of activation when exposed to purified phytoestrogens and are particularly sensitive to coumestrol and genistein. Of the seven commercial diets that were tested, three achieved significant activation on the felid receptors, specifically on ERβ. Across all species, NE—Feline Horse achieved the highest level of activation, which contains added soybean meal for increased protein content (Supplemental Table 2). In SL and CH, NE—Feline Prem Beef achieved the second highest level of activation, and NE—Feline Spec Beef achieved the third. The opposite pattern was observed in ST, in which NE—Feline Spec Beef achieved the second highest level of activation, and NE—Feline Prem Beef achieved the third (Figure 6). Interestingly, NE—Feline Spec Beef contains added soybean meal but NE—Feline Prem Beef does not. The Nebraska manufacturer does not specify the percentage of the total diet that consists of soybean product for those that contain it. Although it is unknown how many samples taken of each diet formulation across facilities came from separate batches, the manufacturer has stated that each lot is made in approximately 2000 lb batches and sells quickly, often selling an entire batch to a single facility at a time [personal communication]. Additionally, the manufacturer states dependability of products by sourcing ingredients from consistent suppliers [personal communication]. Given these statements, it is plausible that the samples we collected over 2 years have most likely spanned multiple lots of each diet but overall should remain similar across lots.

It was intriguing to discover that NE—Feline Prem Beef caused significant activation while lacking a soybean additive, although it is well documented that feedlot-finished cattle consume diets containing high levels of soy [32–34]. This stands to reason that the muscle on these animals may sequester increased concentrations of phytoestrogens compared to animals that only consume more natural forage, such as grass. This was the premise behind including grassfed beef as a negative control in the present study, which was tested in each assay to evaluate if activation could be achieved in the absence of soybean additives both in diet formulation and diet of the source animal prior to slaughter. Grassfed beef did not result in activation of any ERs tested, showing that it contained little to no estrogenic content.

Soybeans contain isoflavone phytoestrogens, most notably, genistein and daidzein, which have been a focus for research on the reproductive success of female herbivores for many years. In more recent years, studies have also begun to investigate the impacts of dietary phytoestrogens on male reproductive success. Previous literature suggests that high levels of phytoestrogens can result in increased weight gain, scrotum circumference, and spermatozoa counts in prepubertal bulls [35, 36]. Despite larger scrotum circumference, limited data also show that phytoestrogens may negatively impact duct epithelium by causing hypertrophy and decrease semen quality by promoting premature capacitation and acrosome loss in bulls and rams [36, 37]. In contrast to the observation in bulls, experiments in rats have demonstrated that a high-phytoestrogen diet results in decreased sperm production and a higher incidence of apoptotic germ cells [22]. These studies demonstrate the potential of dietary phytoestrogens to impact spermatozoa production in a variety of different species. Although no previous studies have investigated this relationship in felids, it could be speculated that they, being carnivores, have not consumed significant levels of phytoestrogens throughout their evolutionary history and, as a result, may be particularly sensitive to them.

Additional studies found that high levels of phytoestrogens cause alterations to gene expression of ERα in the cauda epididymis of male rats and an increase in lipid peroxidation of epididymal sperm [23]. Interestingly, this study by Glover and Assinder did not evaluate changes in gene expression of ERβ, although research has extensively shown that many phytoestrogens have a significantly stronger affinity for ERβ than ERα [20, 24, 36, 38, 39]. Both ERβ mRNA and protein have been identified throughout the efferent ducts and epididymis of feline, mouse, rat, human, and primate male reproductive tracts [24–26]. Furthermore, numerous investigations have identified ERβ in gametic cells throughout spermatogenesis, spermiogenesis, and spermatozoa maturation, while the identification of ERα has been variable depending on study and species, which suggests that ERβ is the predominantly conserved ER found in male germ cells across various species [24, 40, 41]. In humans, both ERα and ERβ have been identified in mature spermatozoa [42, 43], and the importance of estrogen action in the testes and sperm cells has been supported by the discovery of both mRNA and functional ER and aromatase proteins [43–45]. Therefore, the presence of ERs, and particularly ERβ, in the epididymis and gametic cells of other mammals suggests that they may be responsive to estrogenic substances and potential targets of endocrine-disrupting chemicals.

The present data continue to support the findings of an increased affinity of phytoestrogens and other estrogenic compounds for ERβ in three novel species, with all but two of the diet samples tested causing higher receptor activation of ERβ than ERα in SL, ST, and CH. The present data also elucidate a potential relationship between estrogenic diets and spermatozoa defects in a novel species. No prior studies have investigated this phenomenon in tigers, which could contribute to the drastic decline in morphologically normal spermatozoa that has been observed in recent years. Bent midpieces, bent midpieces with retained cytoplasmic droplets, and bent tails were all found to be higher in tigers that were consuming a highly estrogenic diet at the time of semen collection and analysis. Midpiece defects, retained cytoplasmic droplets, and bent tails were also the most frequently recorded defects across prior studies that evaluated morphological integrity in captive tiger spermatozoa over approximately the last 30 years [8, 9, 29].

Another possible implication of exotic felids being fed highly estrogenic diets in managed care could be developmental issues due to fetal exposure. Commercial exotic felid diets have been the primary nutrient source for felids in managed care for decades [18, 19], meaning that captive-born animals would have been exposed to any estrogenic compounds within the diet during gestation. Previous studies have suggested that prenatal exposure to estrogenic compounds in both sexes may lead to reproductive issues such as general reduced fertility in mice and southern white rhinoceros [21, 46] and more specifically, abnormal epididymis development, spermatogenesis [47, 48], and an increase in atretic follicles [49] in rats.

One explanation for poor semen quality in exotic felids has been a reduction in genetic diversity through historical genetic bottleneck events and potential inbreeding in small, managed populations [50, 51]. Although it is known that positive associations have been documented between genetic diversity and semen quality based on pedigree data in the past, recent genomic studies are finding that genetic diversity may not completely account for spermatozoa quality in all exotic felids. In cheetahs, it has been shown that genomic heterozygosity does not correlate with semen quality and is unexpectedly lower in current proven breeders [51]. Furthermore, in tigers, the literature does not support evidence for severe, recent inbreeding in captive populations, even those housed in non-AZA facilities [50]. Therefore, it is plausible that other factors such as dietary differences are contributing to the poor semen quality that is observed.

The potential for dietary estrogenic compounds to cause artificial activation of ERs may be a novel insight into one contribution to morphologically abnormal sperm in captive exotic felids. Our data show that some commercial exotic felid diets are highly estrogenic and result in significant activation of ERs compared to others. We presume that diet estrogenicity is due to the presence of phytoestrogens; however, we did not determine the identity of the estrogenic compounds in this study. Additionally, our data elucidated a correlation between estrogenic diets and tiger spermatozoa defects, suggesting that low estrogenic diets (Milliken—Toronto Horse, AAA—Feline Complete, NE—Feline Prem Horse, and NE—Canine Prem) may be more suitable to feed. Transitioning individuals from high to low estrogenic diets would offer an important opportunity to investigate potential changes more directly in spermatozoa morphology and to continue enhancing our knowledge of possible driving factors of viable sperm production in felids under managed care.

## Supplementary Material

Supp_Figure_1_ioaf161

Supp_Figure_2-Felid_Diet_Study_ioaf161

Supp_Figure_3-Felid_Diet_Study_ioaf161

Supplemental_Table_1-Felid_Diet_Study_ioaf161

Supplemental_Table_2-Felid_Diet_Study_ioaf161

## Data Availability

The data underlying this article are available in NIH GenBank Nucleotide Database and can be found with the unique identification numbers provided in Supplemental Table 1.
