# Supplementary figures and images for: The association between diet estrogenicity in exotic felids and poor spermatozoa quality in tigers (Panthera tigris)
Source: Biol Reprod. 2025 Jul 24;113(3):592–604. doi: 10.1093/biolre/ioaf161 (PMC12448638; doi:10.1093/biolre/ioaf161)

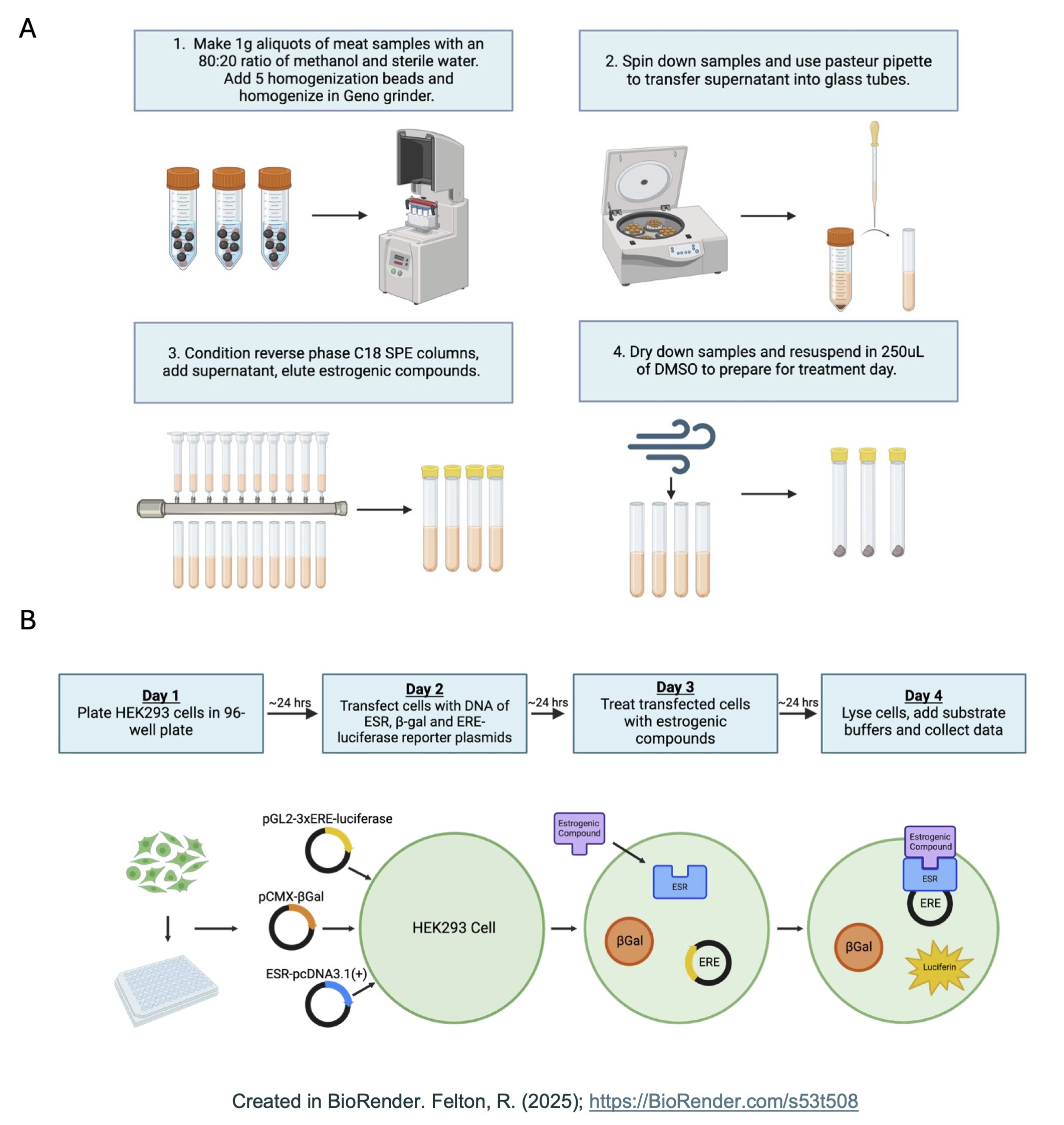

Supplement: Supp_Figure_1_ioaf161 [file supp_figure_1_ioaf161.jpeg]
