# Supplementary material for: The association between diet estrogenicity in exotic felids and poor spermatozoa quality in tigers (Panthera tigris)
Source: Biol Reprod. 2025 Jul 24;113(3):592–604. doi: 10.1093/biolre/ioaf161 (PMC12448638; doi:10.1093/biolre/ioaf161)

A

SLERalpha/1-595 1 MTMTLHTKASGMALLHQIQGNELETLNRPQLKIPLERPLGEVYVDGSKPAVYSYPEGAA 59
STERalpha/1-595 1 MTMTLHTKASGMALLHQIQGNELETLNRPQLKIPLERPLGEVYVDGSKPAVYSYPEGAA 59
CHERalpha/1-595 1 MTMTLHTKASGMALLHQIQGNELETLNRPQLKIPLERPLGEVYVDGSKPAVYNYPEGAA 59

SLERalpha/1-595 60 YDFNAAPAASAPVYGQSGLAYGSGSEAAAFGANGLGGFPPLNSVSPSPLVLLHPPPQLS 118
STERalpha/1-595 60 YDFNAAPAASAPVYGQPGLAYGSGSEAAAFGANGLGGFPPLNSVSPSPLVLLHPPPQLS 118
CHERalpha/1-595 60 YDFNAAPAASAPVYGQSGLAYGSGSEAAAFGANGLGGFPPLNSVSPSPLVLLHPPPQLS 118

SLERalpha/1-595 119 PFLHPHGQQVPYYLENEPSGYAVREAGPPAFYRPNSDNRRQSGRERLASTGDKGSMAME 177
STERalpha/1-595 119 PFLHPHGQQVPYYLENEPSGYAVREAGPPAFYRPNSDNRRQSGRERLASTGDKGSMAME 177
CHERalpha/1-595 119 PFLHPHGQQVPYYLENEPSGYAVREAGPPAFYRPNSDNRRQSGRERLASTGDKGSMAME 177

SLERalpha/1-595 178 SAKETRYCAVCNDYASGYHYGVWSCEGCKAFFKRSIQGHNDYMCPATNQCTIDKNRRKS 236
STERalpha/1-595 178 SAKETRYCAVCNDYASGYHYGVWSCEGCKAFFKRSIQGHNDYMCPATNQCTIDKNRRKS 236
CHERalpha/1-595 178 SAKETRYCAVCNDYASGYHYGVWSCEGCKAFFKRSIQGHNDYMCPATNQCTIDKNRRKS 236

SLERalpha/1-595 237 CQACRLRKCYEVGMMKGGIRKDRRGGRMLKHKRQRDEGEGRNEVGSSGDVRASNLWPSP 295
STERalpha/1-595 237 CQACRLRKCYEVGMMKGGIRKDRRGGRMLKHKRQRDEGEGRNEVGSSGDVRASNLWPSP 295
CHERalpha/1-595 237 CQACRLRKCYEVGMMKGGIRKDRRGGRMLKHKRQRDEGEGRNEVGSSGDVRASNLWPSP 295

SLERalpha/1-595 296 LLIKHTKKNSPALSLTADQMVSALLEAEPPIIYSDYDPSRPFSEASMMGLLTNLADREL 354
STERalpha/1-595 296 LLIKHTKKNSPALSLTADQMVSALLEAEPPIIYSDYDPSRPFSEASMMGLLTNLADREL 354
CHERalpha/1-595 296 LLIKHTKKNSPALSLTADQMVSALLEAEPPIIYSDYDPSRPFSEASMMGLLTNLADREL 354

SLERalpha/1-595 355 VHMINWAKRVPGFVDLSLHDQVHLLECAWLEILMIGLVWRSMEHPGKLLFAPNLLLDRN 413
STERalpha/1-595 355 VHMINWAKRVPGFVDLSLHDQVHLLECAWLEILMIGLVWRSMEHPGKLLFAPNLLLDRN 413
CHERalpha/1-595 355 VHMINWAKRVPGFVDLSLHDQVHLLECAWLEILMIGLVWRSMEHPGKLLFAPNLLLDRN 413

SLERalpha/1-595 414 QGKCVEGMVEIFDMLLATSSRFRMMNLQGEEFVCLKSIILLNSGVYTFLSSTLKSLEEK 472
STERalpha/1-595 414 QGKCVEGMVEIFDMLLATSSRFRMMNLQGEEFVCLKSIILLNSGVYTFLSSTLKSLEEK 472
CHERalpha/1-595 414 QGKCVEGMVEIFDMLLATSSRFRMMNLQGEEFVCLKSIILLNSGVYTFLSSTLKSLEEK 472

SLERalpha/1-595 473 DHIHRVLDKITDTLIHLMAKAGLTLQQQHRRLAQLLLILSHIRHMSNKGMEHLYNMKCK 531
STERalpha/1-595 473 DHIHRVLDKITDTLIHLMAKAGLTLQQQHRRLAQLLLILSHIRHMSNKGMEHLYNMKCK 531
CHERalpha/1-595 473 DHIHRVLDKITDTLIHLMAKAGLSLQQQHRRLAQLLLILSHIRHMSNKGMEHLYNMKCK 531

SLERalpha/1-595 532 NVVPLYDLLLEMLDAHRLHAPANRGGAPMEEMNQSQLATTGSTSAHSLQAYYITEEAGA 590
STERalpha/1-595 532 NVVPLYDLLLEMLDAHRLHAPANRGGAPMEEMNQSQLATTGSTSAHSLQAYYISEEAGA 590
CHERalpha/1-595 532 NVVPLYDLLLEMLDAHRLHAPANRGGAPMEEMNQSQLATTGSTSAHSLQAYYITEEAGA 590

SLERalpha/1-595 591 FPTTV 595
STERalpha/1-595 591 FPTTV 595
CHERalpha/1-595 591 FPTTV 595

B

SLERbeta/1-550 1 MSLCASSHKEFPQLLPIQDMDIKNSPSSLNSPASYNCSQSILPLEHGPIYIPSSYVESR 59
STERbeta/1-550 1 MSLCASSHKEFPQLLPIQDMDIKNSPSSLNSPASYNCSQSILPLEHGPIYIPSSYVESR 59
CHERbeta/1-550 1 MSLCASSHKEFPQLLPIQDMDIKNSPSSLNCPASYNCSQPILPLEHGPIYIPSSYVESR 59

SLERbeta/1-550 60 HEYSAMTFYSPTVMNYGIPSSASNSEGGPGRQTTSPNVLWPTPGHLSPLAIHCQSSLLY 118
STERbeta/1-550 60 HEYSAMTFYSPTVMNYGIPSSASNSEGGPGRQTTSPNVLWPTPGHLSPLAIHCQSSLLY 118
CHERbeta/1-550 60 HEYSAMTFYSPTVMNYGIPSSASNSEGGPGRQTTSPNVLWPTPGHLSPLAIHCQSSLLY 118

SLERbeta/1-550 119 AEPQKSPWCEARSLEPTLPIHRETLKRKASGSSCASPVTSPSSKRDAHFCAVCSDYASG 177
STERbeta/1-550 119 AEPQKSPWCEARSLEPTLPVHRETLKRKASGSSCASPVTSPSSKRDAHFCAVCSDYASG 177
CHERbeta/1-550 119 AEPQKSPWCEARSLEPTLPVHRETLKRKVSGSSCASPVTSPSSKRDAHFCAVCSDYASG 177

SLERbeta/1-550 178 YHYGVWSCEGCKAFFKRSIQGHNDYICPATNQCTIDKNRRKSCQACRLRKCYEVGMVKC 236
STERbeta/1-550 178 YHYGVWSCEGCKAFFKRSIQGHNDYICPATNQCTIDKNRRKSCQACRLRKCYEVGMVKC 236
CHERbeta/1-550 178 YHYGVWSCEGCKAFFKRSIQGHNDYICPATNQCTIDKNRRKSCQACRLRKCYEVGMVKC 236

SLERbeta/1-550 237 GSRRERCGYRVVRRQKSSEEQLRCASKAKKAGGHVTRVKELLLSALSPEQLVLTLLEAE 295
STERbeta/1-550 237 GSRRERCGYRVVRRQKSSEEQLRCASKAKKAGGHVTRVKELLLSALSPEQLVLTLLEAE 295
CHERbeta/1-550 237 GSRRERCGYRVVRRQKSSEEQLRCASKAKKAGGHVTRVKELLLSALSPEQLVLTLLEAE 295

SLERbeta/1-550 296 PPHVLVSRPSTPFTEASMMMSLTKLADKELVHMIGWAKKIPGFVELSLYDQVRLLESCW 354
STERbeta/1-550 296 PPHVLVSRPSTPFTEASMMMSLTKLADKELVHMIGWAKKIPGFVELSLYDQVRLLESCW 354
CHERbeta/1-550 296 PPHVLVSRPSTPFTEASMMMSLTKLADKELVHMIGWAKKIPGFVELSLYDQVRLLESCW 354

SLERbeta/1-550 355 MEVLMVGLMWRSIDHPGKLIFAPDLILDRDEGKCVEGILEIFDMLLATTSRFRELKLQH 413
STERbeta/1-550 355 MEVLMVELMWRSIDHPGKLIFAPDLILDRDEGKCVEGILEIFDMLLATTSRFRELKLQH 413
CHERbeta/1-550 355 MEVLMVGLMWRSIDHPGKLIFAPDLILDRDEGKCVEGILEIFDMLLATTSRFRELKLQH 413

SLERbeta/1-550 414 KEYLCVKAMVLLNSSMHPLAAASQEADSSRKLTHLLNAVTDALVWVIAKSGIPSQQQPV 472
STERbeta/1-550 414 KEYLCVKAMVLLNSSMHPLAAASQEADSSRKLTHLLNAVTDALVWVIAKSGIPSQQQPV 472
CHERbeta/1-550 414 KEYLCVKAMVLLNSSMHPLAAASQEADSSQKLTHLLNAVTDALVWVIAKSGIPSQQQPV 472

SLERbeta/1-550 473 RLANLLMLLSHVRHASNKGMEHLLNMKCKNVVPVYDLLLEMLNAHTLRGHKSLVTGSEC 531
STERbeta/1-550 473 RLANLLMLLSHVRHASNKGMEHLLNMKCKNVVPVYDLLLEMLNAHTLRGHKSLVTGSEC 531
CHERbeta/1-550 473 RLANLLMLLSHVRHASNKGMEHLLNMKCKNVVPVYDLLLEMLNAHTLRGHKSLVTGSEC 531

SLERbeta/1-550 532 SPAEESKSKEGFQNLQSPQ 550
STERbeta/1-550 532 SPAEESKSKEGFQNLQSPE 550
CHERbeta/1-550 532 SPAEESKSKEGFQNLQSPQ 550

C

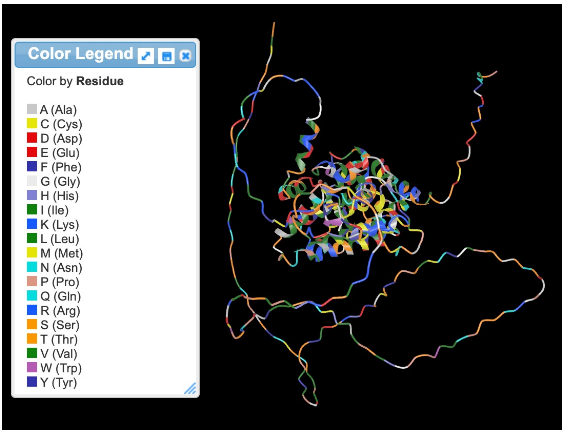

Supplement: Supp_Figure_2-Felid_Diet_Study_ioaf161 [file supp_figure_2-felid_diet_study_ioaf161.pdf]
