# Supplementary material for: The association between diet estrogenicity in exotic felids and poor spermatozoa quality in tigers (Panthera tigris)
Source: Biol Reprod. 2025 Jul 24;113(3):592–604. doi: 10.1093/biolre/ioaf161 (PMC12448638; doi:10.1093/biolre/ioaf161)
Supplement: Supp_Figure_3-Felid_Diet_Study_ioaf161 [file supp_figure_3-felid_diet_study_ioaf161.pdf]

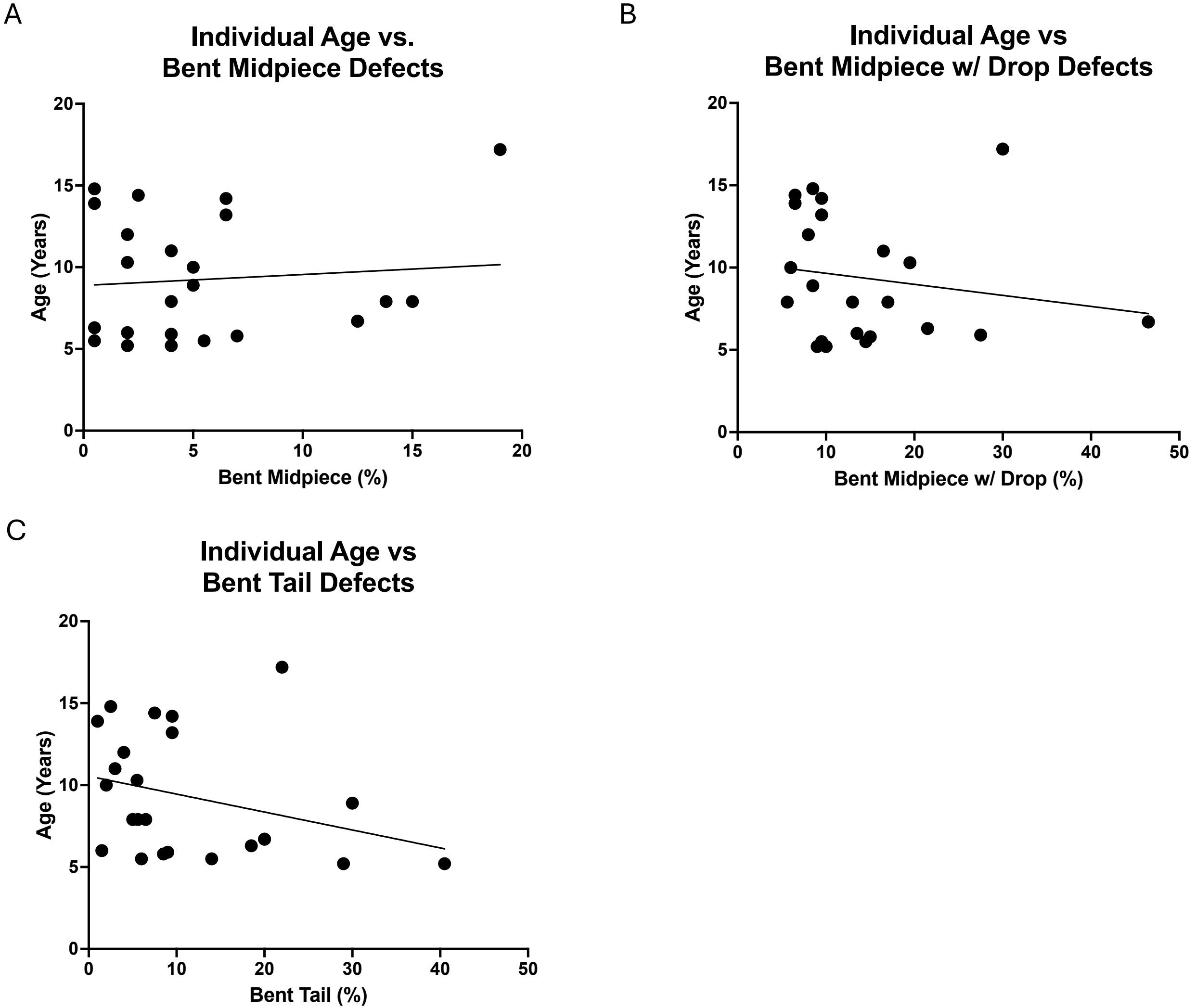
A
Individual Age vs.
Bent Midpiece Defects
Age (Years)
Bent Midpiece (%)
B
Individual Age vs
Bent Midpiece w/ Drop Defects
Age (Years)
Bent Midpiece w/ Drop (%)
C
Individual Age vs
Bent Tail Defects
Age (Years)
Bent Tail (%)
