# Supplementary material for: The association between diet estrogenicity in exotic felids and poor spermatozoa quality in tigers (Panthera tigris)
Source: Biol Reprod. 2025 Jul 24;113(3):592–604. doi: 10.1093/biolre/ioaf161 (PMC12448638; doi:10.1093/biolre/ioaf161)
Supplement: Supplemental_Table_1-Felid_Diet_Study_ioaf161 [file supplemental_table_1-felid_diet_study_ioaf161.docx]

**Supplemental Table 1.** GenBank identifications and primer sequences for ESR1 and ESR2 in the SL, ST, and CH.

|  | **SL** |  | **ST** |  | **CH** |
| --- | --- | --- | --- | --- | --- |
| GenBank ESR1 | PQ834961 |  | PQ827006 |  | PQ827008 |
| GenBank ESR2 | PQ834962 |  | PQ827007 |  | PQ827009 |
| GenBank ESR1 CO | PV164931 |  | PV164932 |  | PV164930 |
| GenBank ESR2 CO | NA |  | PV190972 |  | NA |
| ESR1 F | 5’ – ATGACCATGACCCTCCACAC – 3’ |  | 5’ – ATGACCATGACCCTCCACAC – 3’ |  | 5’ – ATGACCATGACCCTCCACAC – 3’ |
| ESR1 R | 5’ - GGCTTTCCCCACCACAGTCTGA -3’ |  | 5’ - GGCTTTCCCCACCACAGTCTGA -3’ |  | 5’ - GGCTTTCCCCACCACAGTCTGA -3’ |
| ESR2 F | 5’ – ATGTCCCTTTGTGCCTCTTCTCAC – 3’ |  | 5’ – ATGTCCCTTTGTGCCTCTTCTCAC – 3’ |  | 5’ – ATGTCCCTTTGTGCCTCTTCTCAC – 3’ |
| ESR2 R | 5’-TCACTCAGGAGACTGGAGGTTCTGG-3’ |  | 5’-TCACTCAGGAGACTGGAGGTTCTGG-3’ |  | 5’ - CCAGAACCTCCAGTCTCCTCAGTGA – 3’ |
