# Supplementary material for: The association between diet estrogenicity in exotic felids and poor spermatozoa quality in tigers (Panthera tigris)
Source: Biol Reprod. 2025 Jul 24;113(3):592–604. doi: 10.1093/biolre/ioaf161 (PMC12448638; doi:10.1093/biolre/ioaf161)
Supplement: Supplemental_Table_2-Felid_Diet_Study_ioaf161 [file supplemental_table_2-felid_diet_study_ioaf161.docx]

**Supplemental Table 2.** Summary of all diets tested for estrogenic activity and corresponding semen analyses. Including manufacturer, ingredients of diet formulation, if estrogen receptor activation was achieved, if semen collection contained enough spermatozoa for morphological evaluation, and the presence/absence of morphological defects. *Both samples from February 27, 2019 were from the same individual who was consuming two different diets, collected on the same day.

|  |  |  |  |  |  |  |  |  |  |  |
| --- | --- | --- | --- | --- | --- | --- | --- | --- | --- | --- |
| Facility | Diet Name | Ingredients | Activation of ER𝛼 | Activation of ER𝛽 | Collection date | Animals w/ semen analysis | Sufficient spermatozoa to analyze | Bent midpiece defects | Bent midpiece w/ drop defects | Bent tail defects |
| A | Miliken - Toronto horse | Horsemeat, cellulose, tri-calcium phosphate (TZ Feline & Canine Diets only), Toronto Zoo Vitamin-Mineral Premix, Cellulose, Limestone (TZ Small Carnivore only), Vitamin E, Taurine & Flaxseed Screenings. |  |  | March 21, 2019 | 1 | 1/1 | 0/1 | 0/1 | 0/1 |
| B | Nebraska - Feline premium beef | Beef , powdered cellulose, dicalcium phosphate, calcium carbonate, Vitamin Premix (vitamin E supplement, niacin, d-biotin, vitamin A acetate, riboflavin, pyridoxine, folic acid, d-calcium pantothenate, thiamine mononitrate, vitamin D-3 supplement, menadione sodium bisulfate complex - source of vitamin K activity),Trace Mineral Premix (zinc oxide, manganese oxide, copper oxide, mineral oil, sodium selenite, calcium iodate), choline chloride, taurine, salt | X | X | June 27, 2017 | 1 | 1/1 | 0/1 | 0/1 | 0/1 |
| C | Nebraska - Feline premium beef | Beef , powdered cellulose, dicalcium phosphate, calcium carbonate, Vitamin Premix (vitamin E supplement, niacin, d-biotin, vitamin A acetate, riboflavin, pyridoxine, folic acid, d-calcium pantothenate, thiamine mononitrate, vitamin D-3 supplement, menadione sodium bisulfate complex - source of vitamin K activity),Trace Mineral Premix (zinc oxide, manganese oxide, copper oxide, mineral oil, sodium selenite, calcium iodate), choline chloride, taurine, salt |  | X | February 27, 2019 | 1 | 1/1 | 1/1 | 1/1 | 1/1 |
| C | AAA - Feline complete | Beef muscle meat, Kan Tech Feline Complete vitamin/mineral premix. |  |  | February 27, 2019 | 1 | 1/1 | 1/1 | 1/1 | 1/1 |
| D | Nebraska - Feline premium horse | Horsemeat, powdered cellulose, dicalcium phosphate, calcium carbonate Vitamin Premix (Roughage Products, Vitamin E Supplement, Mineral Oil, Niacin Supplement, Biotin, Menadione Sodium Bisulfite Complex  (source of Vitamin K Activity), Vitamin A Supplement, Riboflavin, Pyridoxine Hydrochloride, Folic Acid, Calcium Pantothenate, Thiamine Mononitrate, Vitamin D3 Supplement) Trace Mineral Premix (Copper Sulfate, Manganese Sulfate, Ethylenediamine dihydriodide, Sodium Selenite), Choline chloride, taurine, salt |  |  | June 22, 2017 | 1 | 1/1 | 0/1 | 0/1 | 0/1 |
| E | Nebraska - Feline horse/feline carnivore diet | Horsemeat, meat, meat by-products, fish meal, soy bean meal, dried beet pulp, calcium carbonate, dicalcium phosphate. dried egg, brewers dried yeast, salt, Vitamin Premix (Choline chloride, vitamin E supplement, niacin, vitamin B-12 riboflavin, folic acid, vitamin A acetate, thiamine mononitrate, d-calcium pantothenate, mineral oil, biotin, pyridoxine hydrochloride, vitamin D-3 supplement), taurine, Trace Mineral premix (zinc oxide, manganous oxide, copper oxide, mineral oil, sodium selenite, calcium iodate) |  | X | June 5, 2018 | 1 | 1/1 | 1/1 | 1/1 | 1/1 |
| F | Nebraska - Canine premium | Horsemeat, powdered cellulose, dicalcium phosphate, calcium carbonate Vitamin Premix (Roughage Products, Vitamin E Supplement, Mineral Oil, Niacin Supplement, Biotin, Menadione Sodium Bisulfite Complex (source of Vitamin K Activity), Vitamin A Supplement, Riboflavin, Pyridoxine Hydrochloride, Folic Acid, Calcium Pantothenate, Thiamine Mononitrate, Vitamin D3 Supplement) Trace Mineral Premix (Copper Sulfate, Manganese Sulfate, Ethylenediamine dihydriodide, Sodium Selenite), Choline chloride, taurine, salt |  |  | December 7, 2017 | 2 | 2/2 | 1/2 | 1/2 | 0/2 |
| F | Nebraska - Canine premium | Horsemeat, powdered cellulose, dicalcium phosphate, calcium carbonate Vitamin Premix (Roughage Products, Vitamin E Supplement, Mineral Oil, Niacin Supplement, Biotin, Menadione Sodium Bisulfite Complex (source of Vitamin K Activity), Vitamin A Supplement, Riboflavin, Pyridoxine Hydrochloride, Folic Acid, Calcium Pantothenate, Thiamine Mononitrate, Vitamin D3 Supplement) Trace Mineral Premix (Copper Sulfate, Manganese Sulfate, Ethylenediamine dihydriodide, Sodium Selenite), Choline chloride, taurine, salt |  |  | December 6, 2018 | 2 | 2/2 | 1/2 | 0/2 | 0/2 |
| G | Miliken - Toronto horse | Horsemeat, cellulose, tri-calcium phosphate (TZ Feline & Canine Diets only), Toronto Zoo Vitamin-Mineral Premix, Cellulose, Limestone (TZ Small Carnivore only), Vitamin E, Taurine & Flaxseed Screenings. |  |  | May 10, 2018 | 2 | 2/2 | 2/2 | 1/2 | 0/2 |
| H | Nebraska - Feline premium horse | Horsemeat, powdered cellulose, dicalcium phosphate, calcium carbonate Vitamin Premix (Roughage Products, Vitamin E Supplement, Mineral Oil, Niacin Supplement, Biotin, Menadione Sodium Bisulfite Complex  (source of Vitamin K Activity), Vitamin A Supplement, Riboflavin, Pyridoxine Hydrochloride, Folic Acid, Calcium Pantothenate, Thiamine Mononitrate, Vitamin D3 Supplement) Trace Mineral Premix (Copper Sulfate, Manganese Sulfate, Ethylenediamine dihydriodide, Sodium Selenite), Choline chloride, taurine, salt |  |  | February 22, 2017 | 1 | 1/1 | 1/1 | 0/1 | 0/1 |
| I | AAA - Feline complete | Beef muscle meat, Kan Tech Feline Complete vitamin/mineral premix. |  |  | August 14, 2018 | 1 | 0/1 | NA | NA | NA |
| J | Miliken - Toronto horse | Horsemeat, cellulose, tri-calcium phosphate (TZ Feline & Canine Diets only), Toronto Zoo Vitamin-Mineral Premix, Cellulose, Limestone (TZ Small Carnivore only), Vitamin E, Taurine & Flaxseed Screenings. |  |  | December 4, 2018 | 1 | 1/1 | 0/1 | 1/1 | 1/1 |
| K | Nebraska - Feline premium horse | Horsemeat, powdered cellulose, dicalcium phosphate, calcium carbonate Vitamin Premix (Roughage Products, Vitamin E Supplement, Mineral Oil, Niacin Supplement, Biotin, Menadione Sodium Bisulfite Complex  (source of Vitamin K Activity), Vitamin A Supplement, Riboflavin, Pyridoxine Hydrochloride, Folic Acid, Calcium Pantothenate, Thiamine Mononitrate, Vitamin D3 Supplement) Trace Mineral Premix (Copper Sulfate, Manganese Sulfate, Ethylenediamine dihydriodide, Sodium Selenite), Choline chloride, taurine, salt | X |  | March 22, 2017 | 1 | 1/1 | 0/1 | 1/1 | 0/1 |
| L | Miliken - Toronto horse | Horsemeat, cellulose, tri-calcium phosphate (TZ Feline & Canine Diets only), Toronto Zoo Vitamin-Mineral Premix, Cellulose, Limestone (TZ Small Carnivore only), Vitamin E, Taurine & Flaxseed Screenings. |  |  | June 6, 2018 | 1 | 1/1 | 0/1 | 0/1 | 1/1 |
| M | Miliken - Toronto feline beef | Horsemeat, cellulose, tri-calcium phosphate (TZ Feline & Canine Diets only), Toronto Zoo Vitamin-Mineral Premix, Cellulose, Limestone (TZ Small Carnivore only), Vitamin E, Taurine & Flaxseed Screenings. |  |  | February 28, 2018 | 2 | 2/2 | 1/2 | 1/2 | 1/2 |
| M | Miliken - Toronto horse | Horsemeat, cellulose, tri-calcium phosphate (TZ Feline & Canine Diets only), Toronto Zoo Vitamin-Mineral Premix, Cellulose, Limestone (TZ Small Carnivore only), Vitamin E, Taurine & Flaxseed Screenings. |  |  | January 29, 2019 | 2 | 2/2 | 0/2 | 0/2 | 0/2 |
| N | Nebraska - Feline premium horse | Horsemeat, powdered cellulose, dicalcium phosphate, calcium carbonate Vitamin Premix (Roughage Products, Vitamin E Supplement, Mineral Oil, Niacin Supplement, Biotin, Menadione Sodium Bisulfite Complex  (source of Vitamin K Activity), Vitamin A Supplement, Riboflavin, Pyridoxine Hydrochloride, Folic Acid, Calcium Pantothenate, Thiamine Mononitrate, Vitamin D3 Supplement) Trace Mineral Premix (Copper Sulfate, Manganese Sulfate, Ethylenediamine dihydriodide, Sodium Selenite), Choline chloride, taurine, salt |  |  | July 2022 | 0 | NA | NA | NA | NA |
| N | Nebraska - Canine premium | Horsemeat, powdered cellulose, dicalcium phosphate, calcium carbonate Vitamin Premix (Roughage Products, Vitamin E Supplement, Mineral Oil, Niacin Supplement, Biotin, Menadione Sodium Bisulfite Complex (source of Vitamin K Activity), Vitamin A Supplement, Riboflavin, Pyridoxine Hydrochloride, Folic Acid, Calcium Pantothenate, Thiamine Mononitrate, Vitamin D3 Supplement) Trace Mineral Premix (Copper Sulfate, Manganese Sulfate, Ethylenediamine dihydriodide, Sodium Selenite), Choline chloride, taurine, salt |  |  | July 2022 | 0 | NA | NA | NA | NA |
| N | Nebraska - Feline special beef | Beef , meat, meat by-products, fish meal, soy bean meal, dried beet pulp, calcium carbonate, dicalcium phosphate, dried egg, brewers dried yeast, salt, Vitamin Premix (Choline chloride, vitamin E supplement, niacin, vitamin B-12 riboflavin, folic acid, vitamin A acetate, thiamine mononitrate, d-calcium pantothenate, mineral oil, biotin, pyridoxine hydrochloride, vitamin D-3 supplement), taurine, Trace Mineral Premix, (zinc oxide, manganous oxide, copper oxide, mineral oil, sodium selenite, calcium iodate). |  | X | July 2022 | 0 | NA | NA | NA | NA |
| N | Nebraska - Feline premium horse | Horsemeat, powdered cellulose, dicalcium phosphate, calcium carbonate Vitamin Premix (Roughage Products, Vitamin E Supplement, Mineral Oil, Niacin Supplement, Biotin, Menadione Sodium Bisulfite Complex  (source of Vitamin K Activity), Vitamin A Supplement, Riboflavin, Pyridoxine Hydrochloride, Folic Acid, Calcium Pantothenate, Thiamine Mononitrate, Vitamin D3 Supplement) Trace Mineral Premix (Copper Sulfate, Manganese Sulfate, Ethylenediamine dihydriodide, Sodium Selenite), Choline chloride, taurine, salt |  |  | September 28, 2018 | 4 | 1/4 | 0/4 | 0/4 | 0/4 |
| O | Nebraska - Feline horse/feline carnivore diet | Horsemeat, meat, meat by-products, fish meal, soy bean meal, dried beet pulp, calcium carbonate, dicalcium phosphate. dried egg, brewers dried yeast, salt, Vitamin Premix (Choline chloride, vitamin E supplement, niacin, vitamin B-12 riboflavin, folic acid, vitamin A acetate, thiamine mononitrate, d-calcium pantothenate, mineral oil, biotin, pyridoxine hydrochloride, vitamin D-3 supplement), taurine, Trace Mineral premix (zinc oxide, manganous oxide, copper oxide, mineral oil, sodium selenite, calcium iodate) |  | X | November 14, 2018 | 1 | 0/1 | NA | NA | NA |
| P | Unknown |  |  |  | April 6, 2017 | 1 | 1/1 | 0/1 | 1/1 | 0/1 |
| Q | Nebraska - Feline horse/feline carnivore diet | Horsemeat, meat, meat by-products, fish meal, soy bean meal, dried beet pulp, calcium carbonate, dicalcium phosphate. dried egg, brewers dried yeast, salt, Vitamin Premix (Choline chloride, vitamin E supplement, niacin, vitamin B-12 riboflavin, folic acid, vitamin A acetate, thiamine mononitrate, d-calcium pantothenate, mineral oil, biotin, pyridoxine hydrochloride, vitamin D-3 supplement), taurine, Trace Mineral premix (zinc oxide, manganous oxide, copper oxide, mineral oil, sodium selenite, calcium iodate) |  | X |  |  |  |  |  |  |
| R | Nebraska - Feline premium beef | Beef , powdered cellulose, dicalcium phosphate, calcium carbonate, Vitamin Premix (vitamin E supplement, niacin, d-biotin, vitamin A acetate, riboflavin, pyridoxine, folic acid, d-calcium pantothenate, thiamine mononitrate, vitamin D-3 supplement, menadione sodium bisulfate complex - source of vitamin K activity),Trace Mineral Premix (zinc oxide, manganese oxide, copper oxide, mineral oil, sodium selenite, calcium iodate), choline chloride, taurine, salt |  | X | January 31, 2019 | 2 | 2/2 | 0/2 | 0/2 | 2/2 |
